# Implementation of an online spacing flanker task and evaluation of its test–retest reliability using measures of inhibitory control and the distribution of spatial attention

**DOI:** 10.3758/s13428-023-02327-7

**Published:** 2024-01-16

**Authors:** Sang Ho Lee, Mark A. Pitt

**Affiliations:** 1 Department of Psychology, Seoul National University, Seoul, Korea; 2 Department of Psychology, The Ohio State University, Columbus, OH USA

**Keywords:** Online experiment, Flanker task, Test-retest reliability, Spatial attention, Inhibitory control

## Abstract

The flanker task (Eriksen & Eriksen, Perception & Psychophysics, 16(1), 143-149, 1974) has been highly influential and widely used in studies of visual attention. Its simplicity has made it popular to include it in experimental software packages and online platforms. The spacing flanker task (SFT), in which the distance between the target and flankers varies, is useful for studying the distribution of attention across space as well as inhibitory control. Use of the SFT requires that the viewing environment (e.g., stimulus size and viewing distance) be controlled, which is a challenge for online delivery. We implement and evaluate an online version of the SFT that includes two calibration pretests to provide the necessary control. Test–retest and split-half reliability of the online version was compared with a laboratory version on measures of inhibitory control and measures of the distribution of attention across space. Analyses show that the online SFT is comparable to laboratory testing on all measures. Results also identify two measures with good test–retest reliability that hold promise for studying performance in the SFT: the mean flanker effect (ICC = 0.745) and RTs on incongruent trials across distances (ICC = 0.65–0.71).

**Supplementary Information:**

The online version contains supplementary material available at 10.3758/s13428-023-02327-7.

The flanker task (Eriksen & Eriksen, 1974) has been widely used to measure attentional control of individuals. In the task, a target stimulus must be identified in the presence of adjacent distracting flankers, which disrupt processing of the target. The array of stimuli is congruent if the target and the flankers are mapped onto the same response, and incongruent if they are mapped onto different responses. For example, in an arrow flanker task where the participants should identify the direction of the target arrow as pointing left or right, the array of arrows is congruent when the target and flankers point in the same direction and incongruent when they point in opposite directions (Fig. 1a). The interference caused by flankers (i.e., flanker effect) is measured by the slowdown in response time (RT) or decrease in accuracy on incongruent trials compared to congruent trials. The magnitude of the flanker effect has been used as an index of selective attention, the cognitive ability to inhibit attention to distractors and focus on the target.

**Fig. 1:**
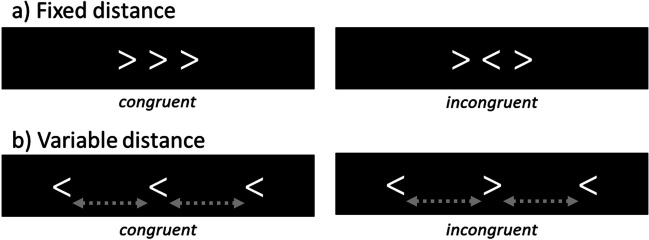
Examples of arrow-flanker stimuli with a fixed distance between the target and the flankers (**a**) and with a variable distance (**b**)

The flanker task is popular (Eriksen & Eriksen, 1974, has been cited over 8000 times) and has been highly influential in advancing our understanding of the mechanisms underlying visual attention (e.g., Botvinick et al., 1999; Lavie et al., 2004; Yantis & Johnston, 1990). Further, the simplicity of the task (e.g., in which direction is the middle arrow pointing) and the fact that most people show a flanker effect, make the task a popular instructional tool (e.g., Diamond et al., 2007; Pozuelos et al., 2019), one that is commonly included in experimentation software packages (e.g., E-Prime, 2016; Inquisit, 2020) and in online experiment platforms such as PsyToolkit (Stoet, 2010), OpenSesame (Mathôt et al., 2012), PsychoPy (Peirce et al., 2019), and Gorilla (Anwyl-Irvine et al., 2020).

Use of the flanker task in a web browser is limited currently to the simplest version of the task in which the flankers are fixed in a single location, immediately adjacent to the target (Fig. 1a). In the spacing flanker task (SFT), which is a variant in which flankers vary in their distance from the target (Fig. 1b), the interference of flankers (i.e., flanker effect) at varying distances is thought to index the amount of attention allocated to each flanker location. When measured at multiple locations, a profile of flanker interference is obtained that describes one’s distribution of attention across space.

Researchers have used the SFT to study the breadth and the shape of the distribution of attention. For example, Eriksen and St. James (1986) found that the size of the attended area can be flexibly adjusted (i.e., zoom lens account) depending on the goal of the task. LaBerge and Brown (1989) described the shape of the attentional distribution by showing that flanker interference gradually decreases as flankers move further from the target (i.e., attentional gradient account). Subsequent work has supported this description of the spatial distribution of attention (e.g., Hübner et al., 2010; Matchock & Mordkoff, 2007; Miller, 1991; Rowe et al., 2007). Other work has found evidence of local suppression of attention around the target (i.e., surround inhibition; Ahmed & De Fockert, 2012; Caparos & Linnell, 2010; Caputo & Guerra, 1998; Lee & Pitt, 2022; Steinman et al, 1995), which makes the distribution of attention non-monotonic (Mexican-hat-shaped; Müller et al., 2005). These findings have inspired computational models that provide mechanistic explanations of how visual processing influences spatial attention (Cutzu & Tsotsos, 2003; Grossberg & Raizada, 2000) and models that describe attention allocation during selective processing of visual stimuli (Weichart et al., 2020; White et al., 2011).

Research using the SFT has also brought out individual differences in the distribution of attention, including developmental changes. Lee and Pitt (2022) reported large differences in the shape of the distribution across individuals, which correlated with working memory capacity. Enns and Girgus (1985; Pasto & Burack, 1997) found that younger children have difficulty adjusting the size of the attended area compared to older children and adults, suggesting that the ability to control the breadth of attention improves with age. In addition, Shalev and Tsal (2003) showed that children with attention deficit hyperactivity disorder (ADHD) exhibit a larger flanker effect than children of the same age when flankers are close to the target. This attentional selectivity in the SFT was also found in older adults as an effect of cognitive aging (Servant & Evans, 2020). Together, these findings show that the SFT can be a useful tool for assessing attentional deficits in multiple populations and for advancing understanding of visual selective attention.

An online version of the SFT would be an asset to the research community because it would expand the potential impact of the task by making it accessible to a broad population of users. However, a significant hurdle of a browser-based SFT is controlling variation in viewing distance and stimulus display characteristics (e.g., size of the monitor, screen resolution). Participants could be using a computer display (laptop or desktop computer) with low or high screen scale (i.e., pixel density per cm) and sit close to the screen or a few feet away. These variables affect the size of the arrows on the retina, which are quantified by the visual angle of the stimuli. The larger the angle, the larger the retinal image of the arrows (Gilinsky, 1951; McCready, 1985). The visual angle must be controlled across participants to measure the distribution of attention accurately. This requirement is challenging because viewing distance and monitor properties are not under the direct control of the experimenter.

We introduce a browser-based version of the SFT. It controls for variation in visual angle by estimating the screen scale and the viewing distance in calibration pretests that precede the SFT. These estimates are then fed into the SFT experiment to control arrow size and distance between arrows across participants. The calibration tests were developed by Li et al. (2020), who showed the test of viewing distance to be reasonably accurate (they did not separately assess the screen scale test). Our use of them serves as an opportunity to evaluate the calibration tests further and to apply the estimated values in a subsequent experiment.

To garner a clear understanding of the reliability of the online SFT, we compared it to a laboratory setup. In the online experiment (reported below), participants completed the task twice (i.e., test and retest) in a place of their own choosing. The laboratory data are from Lee & Pitt (2022, arrow flanker task in Experiment 2; *N* = 158), which were collected in a campus laboratory with precise control of viewing distance, screen scale, and ensured participant engagement by having the experimenter observe the participant during the practice session.

Six measures were used to evaluate reliability (test retest and split half), two pertaining to calibration (screen scale and viewing distance) and four pertaining to the SFT (by-distance and mean flanker effect and by-distance and mean performance on incongruent trials). The consistency of screen scale and viewing distance between test and retest assessed the degree to which the participant’s environment could have contaminated performance in the SFT. The greater the consistency, the greater confidence one can have in the precision of calibration, and by extension the reliability of the online SFT.

With multiple flanker distances, the reliability of performance can be assessed in multiple ways. As discussed in the preceding paragraphs, performance measures in the flanker task have been used for assessing inhibitory control and spatial attention. The magnitude of the flanker effect reflects the level of inhibitory control in selective attention, and the changes in the flanker effect across distances describe how attention is distributed across space.

The distribution of attention across space can be measured by the flanker effect at each distance or by the RT (or accuracy) on incongruent trials at each distance. Precise measurement at each distance is necessary to ensure the estimated profile of attention is trustworthy. The flanker effect has shown moderate to good reliability (correlation coefficient = .4–.91) as measured by Pearson’s *r* or intra-class correlation (ICC; Fan et al., 2002; Hedge et al., 2018; MacLeod et al., 2010; Pronk et al., 2022; Ridderinkhof et al., 2021; Wöstmann et al., 2013; Zelazo et al., 2014). Of most relevance for the current experiment are those conducted online (Luna et al., 2021; Pronk et al., 2022), where the ICC (or Pearson’s *r*) was 0.52–0.63. These values serve as benchmarks for evaluating the reliability of the SFT. The use of RTs on incongruent trials has been suggested as an alternative to the flanker effect because of its relatively high reliability (Chiou & Spreng, 1996; Draheim et al., 2019; Hedge et al., 2018). It is useful when one is interested in *how* response conflict changes across distances (i.e., the distribution of attention) and not in the amount of conflict observed at each distance, which requires knowledge of performance in the congruent condition.

The mean flanker effect (grand mean across distances) served as a summary measure that reflects the average amount of attention across space. We hypothesized that this measure would gauge the general level of inhibitory control. The consistency of the preceding measures between test and retest and across testing environments should provide a comprehensive evaluation of the reliability of the online SFT.

## Methods

The experiments in the current study were approved by the institutional review board of The Ohio State University (ID: 2018B0337).

### Participants

Sixty-one undergraduate students enrolled in introductory psychology at The Ohio State University participated. All participants had self-reported normal or corrected-to-normal vision. The number of participants was determined by a Bayesian power analysis.

### Bayesian power analysis

We aimed to make the lower bound of the 95% confidence interval of intraclass correlation coefficient (ICC; Bartko, 1966) larger than 0.4 (fair reliability; Cicchetti, 1994) when there is a good correlation between test and retest (ICC = 0.7; Cicchetti, 1994). To estimate the power, we randomly sampled the data with 0.7 correlation 10,000 times using code provided by Brysbaert (2019) and calculated the proportion of iterations in which the 95% confidence interval of ICC for the sampled data did not include 0.4. Given the importance of the reliability, we set the desired level of power as 0.95. At least *N* = 57 was required to reach this power level.

### Procedure

Participants received a link to the online experiment to run on their own devices (laptop or desktop only). In two test sessions, they performed three tasks in a browser window in the following order: the credit card task, the blind spot task (Li et al., 2020), and the spacing flanker task. All tasks were controlled by a PsychoJS (online counterpart of PsychoPy; Peirce et al., 2019) script (All code is available at gitlab.pavlovia.org/leearctu/flankeronline). Retest was completed within 3 days of the initial test session.

The methodology was largely the same in the laboratory setting (arrow flanker task in Experiment 2 of Lee & Pitt, 2022). The size (in visual angle), color, shape of the stimuli, distances (in visual angle) between the target and the flankers, stimulus durations, and the viewing distance (50 cm) were the same. Reliability of the laboratory data was assessed by split-half reliability because the data were collected in a single session.

### Credit card task

This task measures pixel density (screen scale) on the participant’s computer screen to determine how many pixels are required to draw an arrow of a given size in the SFT. We used a normalized unit (provided by PsychoJS) instead of pixels to estimate the size that the arrows needed to be on the screen. In the task, the participant has to adjust the image of a student ID card (or credit card) presented on the screen to match the size of their own ID card. The ratio between the width of the card image in the normalized units and the known width of the physical card (85.60 mm; same as a credit card) can be used to determine precisely the size that the arrows must be on the participant’s screen. When finished, participants pressed the keyboard spacebar to proceed to the blind spot task.

### Blind spot task

This task measures viewing distance to the computer screen to determine arrow size and spacing in visual angle. Participants had to close the right eye and fixate the left eye on the cross in the center of the screen. A red circle, whose diameter was adjusted to be 1 cm given the pixel-to-cm ratio obtained in the credit card task, is presented on the left side of the cross. The red circle slowly moves leftward during a trial, and the participants have to press the spacebar when the dot disappears from sight, which indicates the blind spot has been reached. The viewing distance was calculated as D/tan (13.5°), where D is the distance from the fixation (in cm) to where the red dot disappeared.

There were five dot-moving trials in total, with the first two being practice trials. We used the mean of the measured viewing distance in the last three trials to determine the size and the spacing of the arrows to use in the SFT. As a check on the accuracy of measuring viewing distance using the blind-spot task, participants were instructed to keep the viewing distance close to 50 cm in this task and the following flanker task. Viewing distance in the blind-spot task should thus be estimated as close to 50 cm. To help participants measure this distance if they did not have a ruler, a picture in the task instructions illustrated that the sum of the height and the width of a letter-size paper is approximately 50 cm.

### Spacing flanker task

An arrow flanker task similar to Lee and Pitt (2022) was used. At the beginning of a trial, a white fixation cross was presented at the center of the black background. After 500 ms, the cross was replaced by a white target arrow (< or >). On the left and right sides of the target arrow, a white flanker arrow was presented. The target and the flanker arrows pointed in the same (e.g., > > >; congruent) or opposite (e.g., < > <; incongruent) directions. The arrows were displayed for 1000 ms. Participants had to specify the direction of the target arrow within 1500 ms from the onset of the arrows by pressing one of two keyboard keys (“x” indicating the arrow pointed left and “.” Indicating it pointed right). The next trial began after a 1500-ms pause. The size of each arrow was 1.09° × 1.09° in visual angle, which was calculated based on the viewing distance and the screen scale measures in the preceding tasks. The edge-to-edge distance between the target arrow and the flanker arrows was randomly selected among the six flanker distances used in Lee and Pitt (2022): 0.23°, 0.91°, 1.59°, 2.95°, 4.31°, and 5.67°.

 Participants were instructed to respond as quickly and accurately as possible. If they responded incorrectly or did not respond within 1500 ms, they heard a beep and saw a sad face image in the center of the screen. There was a practice block with ten trials, followed by four experimental blocks of 96 randomly ordered trials. Each flanker distance was presented 16 times in each block. The proportion of congruent/incongruent trials was 50% in each block, and the direction of the target arrow was equally likely to be left or right. Participants had a break between blocks for as long as they desired.

## Results

Reliability was measured by the intraclass correlation coefficient (ICC)^1^. Specifically, we used the two-way mixed effects model (ICC (3,1); Shrout & Fleiss, 1979), which is appropriate for assessing the reliability of repeated measurements (Koo & Li, 2016). When calculating the ICC, outliers with exceptionally large differences (> 3SD) between test and retest were excluded from the data because of unusually inconsistent performance across sessions. The credibility of differences among conditions and data sets was assessed using the Bayes factor (BF; Kass & Raftery, 1995) in a Bayesian ANOVA (Wetzels et al., 2012). A BF_10_ below 1 is evidence for the null model (i.e., no effect). The larger the BF_10_, the stronger the evidence for the alternative model. We interpreted the strength of statistical evidence for the alternative model following the classification scheme in Wagenmakers et al. (2018): BF_10_ of 1–3 is anecdotal evidence, 3–10 is moderate evidence, 10–30 is strong evidence, 30–100 is very strong evidence, and > 100 is extreme evidence.

**Fig. 2:**
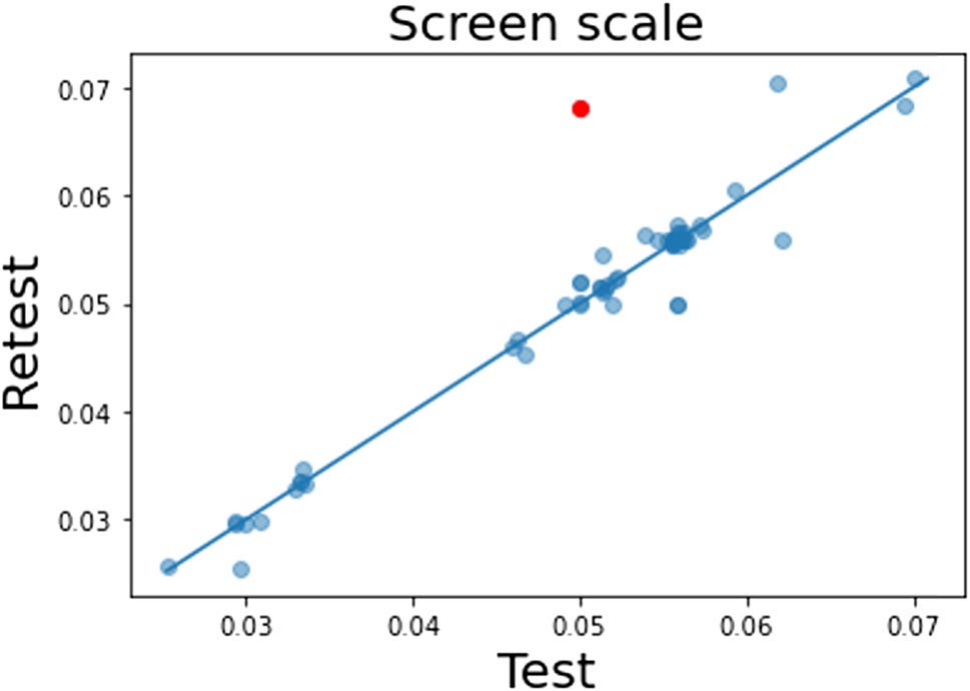
Screen scale measure in test and retest. *Dots* represent participant performance. The trend line displays *y* = *x* and denotes equal performance on test and retest. The *red dot* denotes a participant with a large difference between sessions

We first assessed the test–retest reliability of the screen scale and the viewing distance measures, which is a prerequisite for assessing the reliability of the SFT, in the online data set. Performance in the credit card task is shown in Fig. 2, with the first test session on the *x*-axis and retest on the *y*-axis. The screen scale values in the graph indicate the ratio between the width of the card image in a normalized unit (provided by PsychoJS; Peirce et al., 2019) and the known width of the physical card (85.60 mm). Each dot represents a participant, and most are on or near the diagonal denoting identical performance between sessions. Reliability was high (ICC = 0.981, 95% CI = [0.97,0.99]). These data show that participants adjusted the size of the card in the credit card task as instructed across the two sessions. An outlier (red dot) with an extremely large difference (> 3SD from the mean) between sessions was excluded from the data, as it suggests an inconsistent experiment environment.

**Fig. 3:**
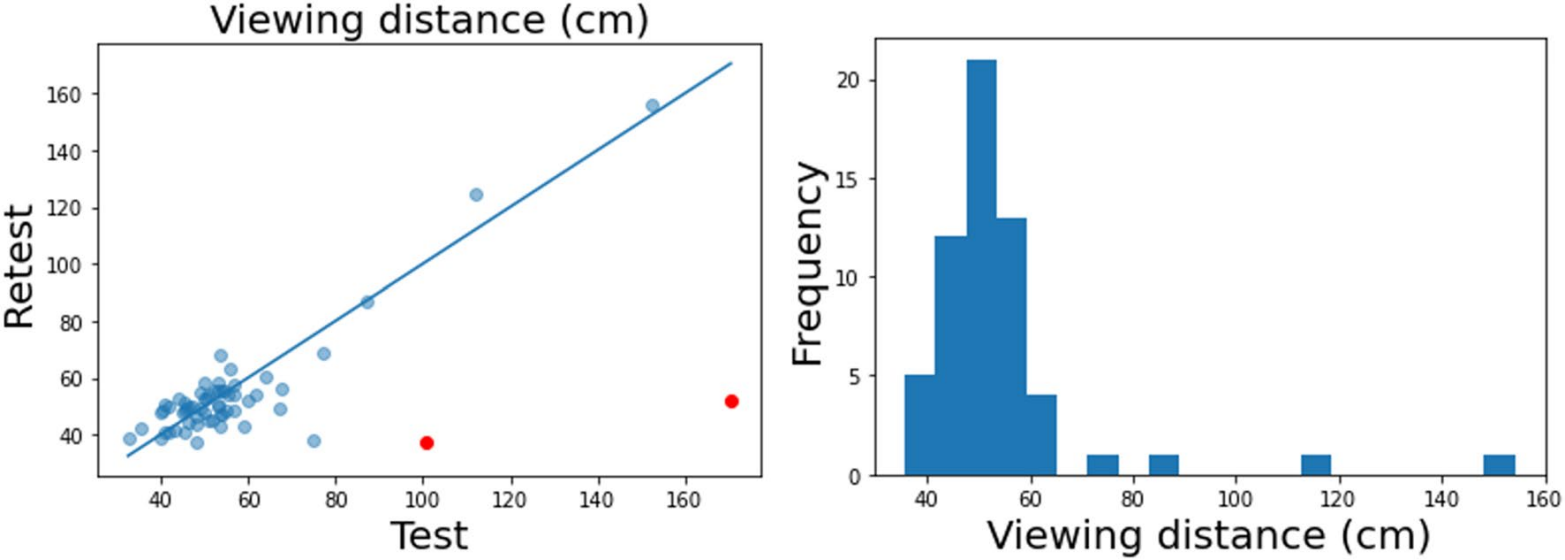
Performance in the blind spot task. The *left panel* shows test–retest reliability for each participant (*dots*), and the *right panel* shows the distribution of the estimated mean viewing distance. The trendline in the left graph displays *y* = *x* function. The red dots denote participants with large differences between sessions

Performance in the viewing distance task (left panel in Fig. 3) also showed excellent reliability (ICC = 0.899, 95% CI = [0.84, 0.94]). Two outliers (red dots) whose viewing distance changed drastically (> 3SD) between test and retest were excluded from analysis. It was unclear whether these outliers changed their viewing distances or made errors in the blind spot task. However, as both outliers showed much larger viewing distance in the test session than in the retest session, they may not have fully understood the instructions in the first session. After performing the task again during retest, viewing distances are more in line with other participants. To ensure accurate calibration, researchers may consider redirecting participants to the instruction screen and reassessing the viewing distance when the estimated viewing distance deviates greatly from the viewing distance recommended in the instruction.

Recall that we asked participants to maintain a 50-cm viewing distance as a means of assessing the accuracy of estimating viewing distance. The right panel of Fig. 3 contains a histogram of estimated viewing distance, which clusters around 50 cm for most participants, with the mean viewing distance being 54 cm. These results show that the blind spot task approximates viewing distance well.

The preceding analyses demonstrate that the calibration tasks are accurate and reliable, paving the way for investigating the reliability of the SFT. As mentioned above, analyses of task performance focused on measures that map the spatial distribution of attention (by-distance flanker effect in RT/Accuracy and by-distance RT/Accuracy on incongruent trials) and measures of inhibitory control (mean flanker effect in RT/Accuracy and mean RT/Accuracy on incongruent trials).

**Fig. 4:**
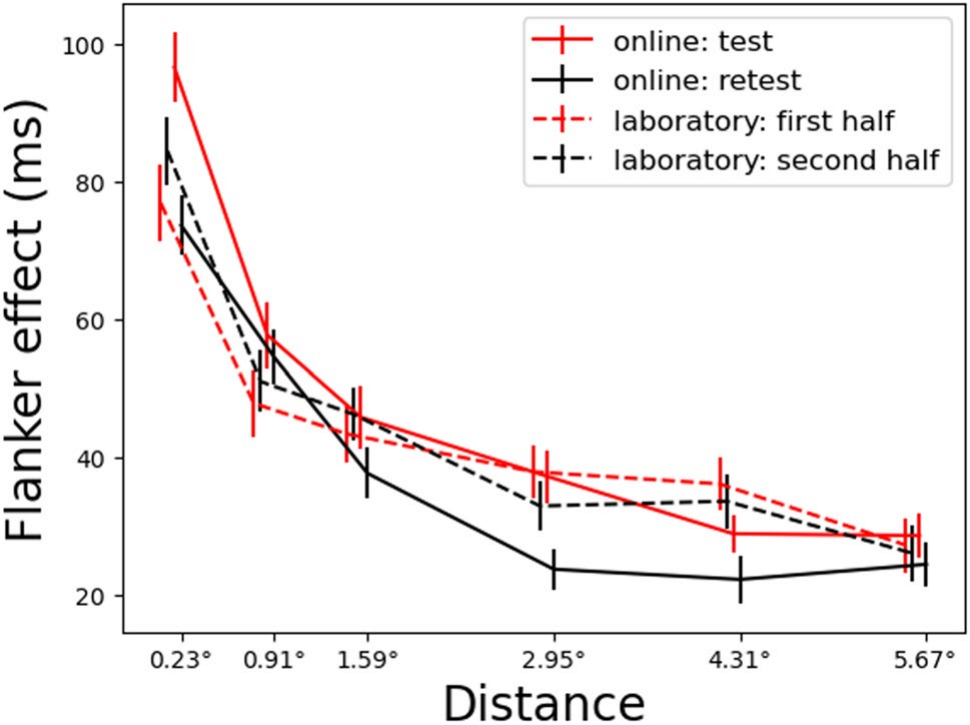
Mean flanker effect across distances. The *error bars* indicate the standard errors of the means. Positions of the lines were adjusted slightly to avoid overlap

Figure 4 contains the flanker effect in RT across the six distances in the online and laboratory data sets, showing a typical gradient pattern of smaller effects at farther distances. In all data sets, evidence for the flanker effect was strong at all distances (all BF_10_s > 100; see Supplement S1 for the details of BF calculations, comparisons among distances, and the flanker effect in accuracy). A robust flanker effect and a gradient pattern across distances are signature outcomes in the laboratory version of the SFT (e.g., Hübner et al., 2010; LaBerge & Brown, 1989; LaBerge et al., 1991), demonstrating that performance in the online SFT is qualitatively similar to its lab-based counterpart.

Statistical comparisons using Bayesian two-way ANOVAs supported the similarity in performance between the online and laboratory SFT. We compared the full laboratory data with the online test session data to exclude the influence of the practice effect between test and retest in the online data set, which is discussed in the following subsection. The two-way ANOVA models examined the effects of the distance and the task type (online or laboratory) on the four measures: flanker effect in RT and accuracy, and the RT and accuracy on incongruent trials. The task type did not show statistical evidence for the main effect on all measures (BF_10_s < 2.12) except for the accuracy on incongruent trials (BF_10_ > 100). There was strong evidence for the main effect of the distance (BF_10_s > 100) on all measures, but no evidence for the interaction effect between the distance and the task type (BF_10_s < 0.4) on all measures. These results suggested that the online and laboratory SFT similarly captured the measures of interest and their changes across distances.

## Reliability of measures of spatial attention and inhibitory control

Reliability of the online SFT was assessed in two ways. The first was to calculate test–retest reliability using the ICC. The second involved comparing reliability with that in the laboratory task. To make this second comparison, we assessed split-half reliability across blocks^2^. For the laboratory experiment, this consisted of a single comparison of blocks 1 and 2 because there was one test session with two blocks. For the online experiment, the split-half analysis was performed twice, once between the first two blocks in the test session and again between the first two blocks in the retest session. The results across the four measures of interest (by-distance flanker effect, mean flanker effect, by-distance performance on incongruent trials, and mean performance on incongruent trials) are shown in Table 1. Each measure was derived from both RT and accuracy data.

**Table 1:** Reliability of the flanker effect and responses on incongruent trials. The values in the tables are intraclass correlation coefficients (ICC)

Flanker distance	Flanker effect				Incongruent trials			
	Test–retest	Split-half			Test–retest	Split-half		
	Online	Online:Test	Online: retest	Laboratory	Online	Online:Test	Online: retest	Laboratory
A. Accuracy								
Mean	0.531	0.65	0.258	0.419	0.582	0.62	0.355	0.509
0.23°	0.455	0.196	0.513	0.315	0.497	0.199	0.408	0.39
0.91°	0.04	0.198	0.082	0.221	0.223	0.175	0.217	0.249
1.59°	0.398	0.138	0.128	0.137	0.513	0.207	0.047	0.147
2.95°	0.275	0.025	0.084	0.26	0.408	– 0.084	– 0.023	0.497
4.31°	0.124	0.521	0.174	0.244	0.406	0.536	0.34	0.325
5.67°	0.281	0.076	0.525	0.2	0.335	0.021	0.346	0.224
B. RT								
Mean	0.745	0.565	0.562	0.626	0.717	0.807	0.791	0.853
0.23°	0.524	0.359	0.393	0.595	0.674	0.624	0.656	0.771
0.91°	0.604	0.324	0.453	0.362	0.714	0.672	0.677	0.686
1.59°	0.447	0.212	0.564	0.428	0.662	0.553	0.662	0.769
2.95°	0.238	0.077	0.146	0.384	0.652	0.654	0.649	0.745
4.31°	0.104	0.141	0.137	0.289	0.687	0.681	0.589	0.713
5.67°	0.484	0.056	0.139	0.265	0.65	0.713	0.565	0.673

We begin by focusing on the ICCs of the mean measures shown in the top row of panels A (accuracy) and B (RT). Looking first at the accuracy data, ICC is variable in both the flanker effect and performance on incongruent trials, ranging from 0.258 to 0.65. Low between-participant variability (SD: 0.04–0.05) and very high accuracy (> .974 across experiments) conspire to cause the variability. ICCs in these circumstances are highly sensitive to non-systematic changes in responding between sessions. When almost all participants perform close to ceiling, a few who perform inconsistently between sessions can dramatically alter ICC (see the scatterplots in section S3 of the Supplement). This variation explains why ICCs are so low in the retest session of the online SFT compared to the test session.

Turning to the reliability of the mean RT data, overall, they show greater reliability than the accuracy data across environments (only one of the eight means is lower), with the ICCs for the mean measures ranging from 0.562 to 0.853. In the flanker effect data, ICCs are somewhat lower in the online compared to the laboratory setting. In the incongruent-trials data, ICCs are much higher than those for the flanker effect; they are also similar across the two environments.

Inspection of ICCs across the six distances shows much greater variability, both in accuracy and in RT. The accuracy data exhibit a wide range of ICC values (– 0.084 to 0.536) with no systematic patterns across distances or across testing environments. The small sample of observations at each distance (1/6 of the total number of trials) appears to make accuracy estimation highly sensitive to random errors, resulting in low and unstable ICCs. In contrast, the ICCs for the by-distance RT exhibit greater consistency across test environments. In the test–retest comparison, the ICCs for the by-distance flanker effects at close distances (0.23–1.59°) are comparable (range, 0.447–0.604) and similar to the reliability obtained in other online flanker tasks with a fixed flanker location (range, 0.52–0.63; Pronk et al., 2022; Luna et al., 2021). However, the flanker effects at further distances (2.95–5.67°) are much less reliable (0.104, 0.238), except for a relatively high test–retest ICC (= 0.484) at 5.67° in the online data set. The laboratory data set also shows a declining trend in ICCs with increasing distances, from 0.595 at 0.23° to 0.265 at 5.67°, although the ICCs are not as variable as in the online setting. Split-half ICCs in the online data set exhibit a similar trend.

Compared to the flanker effect data, reliability is greater in the RT data on incongruent trials. This is true across all environments and distances. Test–retest reliability of the online SFT ranges from 0.65 to 0.71 across distances. When split by session (middle two columns), reliability suffers only marginally. Comparison with the laboratory data shows that laboratory ICC tends to be superior at the closer distances and comparable at the furthest distances. Overall, consistently high ICCs in the RT data suggests that RT is a more reliable measure of individual differences than accuracy in the flanker task. Further, that this consistency is best for the incongruent RT data across distances suggests that they are a viable alternative for measuring performance across distances (Draheim et al., 2019).

**Fig. 5:**
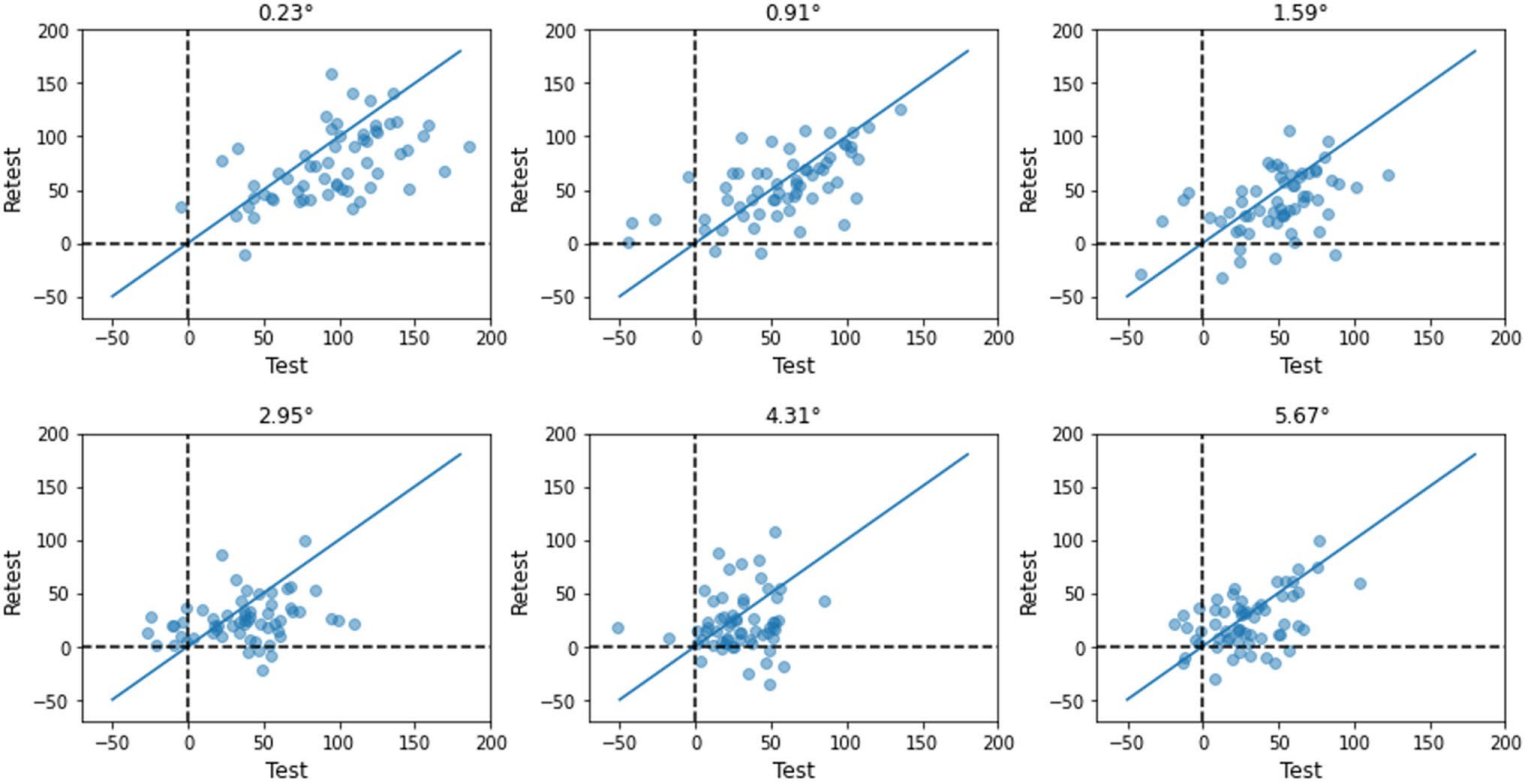
Scatterplots of the flanker effect (ms) comparing test with retest at each distance. *Dotted lines* indicate zero flanker effects. The blue lines are *x* = *y* lines

What explains the low reliability of the RT flanker effect at far distances? The answer lies in minimal variability between participants (as noted above) and relatively large practice effects. Figure 5 shows the variability of the flanker effect among participants and between test and retest across distances in the online data set. Note that variability in test and retest drops as distance increases, and practice effects, visible as points below the diagonal blue lines, tend to shrink as well (see Supplement S4 for the variability and practice effect measures). At distances 4.31° and 5.67°, the flanker effects cluster around small values, with the standard error of the mean being the smallest (2.7–3.3 vs. 3.8–5.1 ms at other distances). Low variability is a major obstacle to reliability because the reliability of a measure becomes zero if there is no variation across participants (Hedge et al., 2018). Further, small individual variation in the flanker effect at far distances could be in part due to a floor effect. At these distances, the flanker effect itself is small, with a floor of zero (dotted lines in Fig. 5), although participants occasionally show a reverse flanker effect. Although less extreme, the floor effect is also visible in the laboratory data (see Supplement S5 for the scatterplots), reinforcing this account of the low reliability at far distances.

Across test and retest there is occasional evidence of a practice effect. When the flanker effect is small, a practice effect (i.e., retest) can lead to a floor effect, reducing reliability. In the online data set (Fig. 4), the flanker effect tends to be smaller in the retest, suggesting that participants learned to ignore distractors with practice (e.g., Brown & Fera, 1994; Kelly & Yantis, 2009). However, statistical comparisons between test and retest at each distance show that the evidence supporting a practice effect is selective. Evidence is strong at 0.23° (BF_10_ > 100), moderate at 2.95° (BF_10_ = 4.6), and very weak at the remaining distances (all BF_10_s < 2). The laboratory data set did not show evidence for practice effects at any distance (all BF_10_s < 0.13).

Practice effects are largest at 0.23° and 2.95° in the online data set, yet reliability in the former is twice that of the latter. At 2.95°, practice effects reduce the flanker effect in the retest toward zero (note that variability is lower in retest than test), reducing the variability among participants. By contrast, the flanker effect at 0.23° rarely hit floor even with a noticeable practice effect. These observations suggest that to minimize the influence of practice and floor effects on reliability, the flanker effect should be sufficiently large. Implementation of experimental manipulations reported to increase the flanker effect (e.g., presenting the target in random locations; de Souza Almeida et al., 2021) might prove effective in addressing this issue.

This in-depth by-distance analysis shows that the flanker effect can easily be unreliable depending on the magnitude of the flanker effect, degree of individual variability, and practice effects. These problems come to light in the SFT because of the multiple flanker distances. The variability in ICC across distances suggests that the ICCs for the mean measures are more stable and thus reliable. The mean flanker effect would be less affected by random errors than the flanker effect at each distance. The current data support this hypothesis. As a single metric that reflects the level of inhibitory control, the mean flanker effect is more reliable than the flanker effect at any of the single distances in Table 1. The test–retest reliability of the mean flanker effect (0.745) is greater than that typically found in the fixed-distance flanker effect (e.g., 0.4–0.57 in Hedge et al., 2018; 0.55–0.63 in Pronk et al., 2022).

## Reliability of measures of the shape of the attentional distribution

The preceding analysis focused on individual measures of inhibitory control without considering the shape of the attentional distribution. Since the SFT is mainly used to map the distribution of attention, the reliability of measures that capture the shape of the distribution is also of interest. A challenge in developing such a measure is that the attentional distribution is not the same across individuals. Although the aggregate data in Fig. 4 show a gradient pattern in which the flanker effect monotonically decreases with distance, at an individual level, the flanker effect pattern is frequently non-monotonic and shows substantial variation across individuals (Lee & Pitt, 2022). Further, the practice effects in the current data suggest that the shape of the attentional distribution could vary across sessions, diminishing its reliability.

We roughly inferred the functions that describe the shape of the attentional distribution by fitting a simple linear function to the flanker effect in RT (and RTs on incongruent trials) across distances. The function is defined as $\left(x\right)={a}_{0}+{a}_{1}x$ , where $x$ is the distance, ${a}_{0}$ is a constant term that reflect the general magnitude of the flanker effect (or RT), and ${a}_{1}$ is a slope parameter that measures the changes in the flanker effect across distances. This function is simplistic, but represents a conventional assumption about the attentional distribution, whether it is monotonic or non-monotonic, that the amount of attention peaks at the target location and gradually diminishes with the distance from the target (e.g., LaBerge & Brown, 1989; Müller et al., 2005). Estimates of ${a}_{1}$ for both the flanker effect and RTs on incongruent trials were smaller than zero in all data sets (all BF_10_s > 100), with the mean values ranging between – 12.26 and – 6.72. This suggests that ${a}_{1}$ effectively represents the declining trend (i.e., negative slope) of the flanker effect (or RTs on incongruent trials) across distances.

Table 2 shows the ICCs for the estimates of the free parameters ${a}_{0}$ and ${a}_{1}$. The ICCs are generally higher for the functions derived from RTs on incongruent trials than the functions derived from the flanker effect, with the mean difference in the ICC being 0.18. This result is in line with the higher reliability of by-distance RTs on incongruent trials compared to by-distance flanker effects in Table 1. Note that the reliability of the functions is inherently constrained by the reliability of the by-distance measures.

**Table 2:** ICC values for the parameters in the linear functions

Parameter	Flanker effect in RT				Rts on incongruent trials			
	Test–retest	Split-half			Test–retest	Split-half		
	Online	Online: Test	Online: retest	Laboratory	Online	Online:Test	Online: retest	Laboratory
${a}_{0}$	0.632	0.48	0.437	0.541	0.701	0.717	0.755	0.797
${a}_{1}$	0.404	0.038	– 0.081	0.333	0.453	0.244	0.062	0.473

The constant term ${a}_{0}$ is consistently more reliable than ${a}_{1}$ in all functions. This is reasonable considering the high reliability of the mean measures compared to by-distance measures (Table 1). Using RTs on incongruent trials made ${a}_{1}$ moderately reliable (ICC > 0.4) in the online test–retest and laboratory split-half analyses, suggesting that ${a}_{1}$ can be a practical measure for assessing the shape of the attentional distribution in some circumstances. However, ${a}_{1}$ in the online data set is much less reliable in the split-half analysis. This suggests that having a sufficient number of trials is particularly important when assessing the shape of the attentional distribution using the SFT.

Limited reliability of the simple linear model might be attributed to large individual differences and non-linear shapes of the attentional distribution reported in past studies (e.g., Lee & Pitt, 2022). However, using higher-order polynomial functions (e.g., quadratic and cubic functions) that can describe non-linear shapes did not improve the reliability of the parameters. We suspect they are overly sensitive to non-systematic changes (e.g., random errors) in the flanker effect (or RTs on incongruent trials) patterns.

A further challenge in modeling performance across distances is that there are systematic changes in the shape of the attentional distribution between test and retest (e.g., practice effect). In the online data set, the value of ${a}_{1}$ in the function of RTs on incongruent trials is closer to zero in the retest for 77% of participants. This is in line with the data in Fig. 4, where the retest (solid black) function is flatter relative to the test (solid red) function, mainly due to the relatively large decrease in the flanker effect (or RTs on incongruent trials) at the closest distance. During the first session, participants appear to learn to suppress the most distracting (closest) flankers, or distribute attention evenly across space, to improve performance, which then lowers the value of ${a}_{1}$ during retest. The laboratory data set, although also exhibiting variability across sessions, do not display such systematic changes in the sample, suggesting that online experiments are prone to practice effects. Together, these observations suggest that more robust models are needed to reliably describe and quantify the shape of the attentional distribution.

## Discussion

Online experimentation has become an accepted platform for data collection. A longstanding concern about the lack of experimental control has limited the use of tasks that require calibration, such as presenting stimuli at specific visual angles (Angele et al., 2022; Grootswagers, 2020). Methods to address this limitation have recently been introduced (Brascamp, 2021; Li et al., 2020), and the current study capitalized on them to evaluate the viability of an online SFT.

The flanker task is one of a handful of congruency tasks (e.g., Stroop task; Stroop, 1935, Simon task; Simon, 1969) in which goal-irrelevant information interferes with the response to the target. Congruency tasks commonly measure inhibitory control, with the size of the congruency effect (i.e., performance difference between congruent and incongruent conditions) assessing the ability to suppress responses to irrelevant stimuli (e.g., flankers). However, mapping the distribution of attention is a distinct use of the flanker task. Unlike other congruency tasks, the flanker task is inherently a test of spatial attention because goal-relevant (i.e., target) and goal-irrelevant (i.e., flanker) stimuli are presented at different locations. That is, the interference of flankers in target processing depends on the distribution of attention around the target. This property of the flanker task makes it suitable for mapping the spatial distribution of attention. An accurate profile of spatial attention in the flanker task can be used to improve our understanding of selective attention across space, as well as provide additional measures of attentional control.

The reliability of multiple performance measures of the SFT were evaluated to determine which are trustworthy. Reliability of the flanker effect at each distance was moderate and inconsistent, with some distances showing especially low reliability, due in part to practice effects and low participant variability. In contrast, the mean flanker effect yielded good reliability (test–retest ICC = 0.745 in the online task), better than that found with the single-distance version of the task conducted online (0.52–0.63; Pronk et al., 2022; Luna et al., 2021). Higher ICCs of the mean measure can be due to a larger number of trials compared to by-distance measures (Hedge et al., 2018; Ishigami and Klein, 2010). However, the comparison with the ICCs from fixed-distance flanker tasks with comparable total number of trials (e.g., 320 trials in Pronk et al., 2022 vs. 384 trials in the current SFT) shows that the increase in ICC is not solely because of a larger number of trials. The ICCs for the mean flanker effect in the SFT was comparable to those in the fixed-distance flanker tasks even when the number of trials were much smaller (ICC = 0.56–0.57 in the split-half analysis using only 96 trials).

Like the traditional flanker task, the mean flanker effect in the SFT is another index of the degree of inhibitory control, but one that is less influenced by how attention is distributed across distances or at any one distance. When the flanker effect decreases smoothly across distances, as in Fig. 4, the flanker effect at a fixed distance may provide a good estimate of inhibitory control. When the function is non-monotonic (e.g., Ahmed & De Fockert, 2012; Caparos & Linnell, 2010; Lee & Pitt, 2022), however, the flanker effect at a specific flanker location might be much less reliable. The reason for this is that a small change in the breadth of the attentional distribution between test and retest can drastically change the amount of attention (flanker effect) at a certain location in a non-monotonic distribution. For example, non-monotonic functions often have a local minimum at a distance near the target. If this distance changes from test to retest by even a small amount (e.g., from 0.91° to 1.59°), reliability could drop substantially at multiple distances. Knowledge of the shape of an individual’s distribution of attention would provide guidance on which derived measure to trust most.^3^

When mapping the distribution of attention, we must rely on by-distance measures even if they are not as reliable as the mean measures. RT in the incongruent condition at each distance is a promising alternative measure for improving the reliability of the attentional distribution mapped by the SFT. This measure showed better and more consistent reliability (0.55–0.77) across distances than the flanker effect in RT (0.06–0.6). As discussed above, this is likely a result of using a direct performance measure rather than an indirect (subtracted) measure. To the extent one is interested in measuring the distribution of attention, use of the RT profile on incongruent trials would be preferable.

More challenging is a robust measure of the shape of the attentional distribution. The reliability of the slope parameter in a simple linear function was moderate at best (ICC > 0.4). This parameter might serve as a rough measure of the attentional distribution in the SFT, although it cannot describe non-monotonic shapes of the attentional distribution. Development of a cognitive model that characterizes the shape of the attentional distribution might prove a more promising approach (e.g., Cutzu & Tsotsos, 2003; Grossberg & Raizada, 2000). A theoretically based model could provide reliable parameters that reflect individual differences in the shape of the attentional distribution, rather than just describing observed patterns (e.g., polynomial functions).

Comparison of the online and laboratory data sets consistently suggested that performance in the online SFT is similar to that in the laboratory SFT. The measures used in the current study rarely showed statistical evidence for differences between experiment environments. A noticeable difference was that practice effects were evident only in the online data set. This difference could be minimized by having more practice trials, as participants learn to reduce the flanker effect mainly in early trials (Ishigami & Klein, 2011; Ishigami et al., 2013). The systematic changes observed in some measures (i.e., flanker effect, linear slope parameter) between sessions in the online data set suggest that minimizing practice effects may enhance the reliability of the online SFT.

Finally, we replicated and extended Li et al. (2020), showing that the calibration pretests reliably estimated the two environmental variables necessary to ensure the flanker task is standardized across participants: pixel density of the computer screen and viewing distance to the screen. Test–retest reliability of both variables was high, with ICCs being close to or greater than 0.90. This finding reduces the concern about uncontrolled experimental environment for online tasks that require precise control over viewing distance and stimulus size.

In summary, we show that an online SFT is feasible, due in large part to the good precision and reliability of the calibration pretests. We also show that the online SFT can be a reliable task for studying spatial attention and inhibitory control, although researchers should be cognizant of possible practice effects. Unlike fixed-distance flanker tasks that typically rely on a single measure (i.e., flanker effect), the SFT provides multiple informative measures while keeping the task simple (respond only to the target object). RTs on incongruent trials and the mean flanker effect across distances, which have not been commonly used in previous studies, hold promise as novel and reliable measures that build on the widely used fixed-distance flanker effect.

### Supplementary Information

Below is the link to the electronic supplementary material.

Supplementary file1 (DOCX 290 KB)

## Footnotes

1 ICC reflects both correlation and agreement between measurements at two different time points. This is a more desirable measure of reliability than other measures that only reflect correlation (e.g., Pearson correlation coefficient) or agreement (e.g., paired *t* test).

2 A correction (Brown, 1910; Spearman, 1910) has been proposed to predict the ICC of a longer test when there is a small number of observations per condition (split-half data). Analyses using corrected values are reported in section S2 of the Supplement for the split-half data only. The correction increases the ICC by an average of 0.24 points across conditions. We chose to be conservative in interpreting the associations and relied on the uncorrected values when interpreting the results.

3 The distribution of attention can be non-monotonic at an individual level even though the flanker effect monotonically decreases in the aggregate data. See Lee & Pitt (2022).
