# Supplementary material for: Implementation of an online spacing flanker task and evaluation of its test–retest reliability using measures of inhibitory control and the distribution of spatial attention
Source: Behav Res Methods. 2024 Jan 16;56(6):5947–58. doi: 10.3758/s13428-023-02327-7 (PMC11335792; doi:10.3758/s13428-023-02327-7)
Supplement: Supplementary file 1 — Supplementary file1 (DOCX 290 KB) [file 13428_2023_2327_MOESM1_ESM.docx]

**Supplement**

S1. Bayes factor calculation for the flanker effect across distances.

The Bayes factors were calculated by comparing the likelihoods of two linear models: null model and alternative model. The null model assumed that the RT did not differ between congruent and incongruent trials or among distances. The alternative model had an additional parameter to explain the differences in RT between congruent and incongruent trials or the differences in the flanker effect across distances. We followed Wetzels et al. (2012) for model specification. For the calculation of model likelihood, we sampled four chains of 20000 samples using a sequential Monte Carlo sampler in PyMC3 (Python package; Salvatier et al., 2016).

Statistical evidence was strong for the changes in the flanker effect across distances (BF_10_ > 100 in both test and retest). Pairwise comparisons of the flanker effect between two distances in the online-only test session data showed that the flanker effect at the closest distance (0.23°; 99ms) was larger than those at all other distances (BF_10_s > 100). The flanker effects at 0.91° (59ms) and 1.59° (50ms) were larger than those at 4.31°-5.67° (BF_10_s > 22.25). No statistical evidence was found for the differences in the flanker effect across 2.95°-5.67° (BF_10_s < 0.66; 29-38ms). The laboratory data set showed similar changes in the flanker effect across distances.

The flanker effect in accuracy was more variable than that in RT. Statistical evidence for the flanker effect was strong at 0.23°-1.59° in all data sets (BF_10_s > 44.22), but inconsistent at far distances, with BF_10_ ranging between 0.18 and >100. The online retest data consistently showed weak statistical evidence for the flanker effect in accuracy at 2.95°-5.67° (BF_10_s < 1.88).

S2. ICCs corrected by the Spearman-Brown prediction formula.

The Spearman-Brown prediction formula predicts how changing the length of the test affects its reliability. The formula is defined as $r_{SB}= nr/(1+\left( n-1 \right)r)$, where $r$ is the reliability (ICC) and $n$ is the ratio of the new test length to the original test length. To predict the test-retest reliability with four blocks per session (online TRT) using the split-half data with one block per session, the Spearman-Brown prediction with $n$ = 4 was applied to the split-half ICCs in Tables 1 and 2 in the main text. Tables S1 and S2 show the predicted ICCs. They are higher than the uncorrected ICCs in Tables 1 and 2 (in the main text) overall, sometimes much higher.

Table S1. Split-half ICC values predicted by the Spearman-Brown prediction formula for the flanker effect and responses on incongruent trials.

A. Accuracy

| Flanker distance | Flanker effect | | |  | Incongruent trials | | |
| --- | --- | --- | --- | --- | --- | --- | --- |
|  | split-half (corrected) | | |  | split-half (corrected) | | |
|  | online:  test | online: retest | laboratory |  | online:  test | online: retest | laboratory |
| Mean | 0.881 | 0.582 | 0.743 |  | 0.867 | 0.688 | 0.806 |
| 0.23° | 0.494 | 0.808 | 0.648 |  | 0.498 | 0.734 | 0.719 |
| 0.91° | 0.497 | 0.263 | 0.532 |  | 0.459 | 0.526 | 0.57 |
| 1.59° | 0.39 | 0.37 | 0.388 |  | 0.511 | 0.165 | 0.408 |
| 2.95° | 0.093 | 0.268 | 0.584 |  | -0.449 | -0.099 | 0.798 |
| 4.31° | 0.813 | 0.457 | 0.564 |  | 0.822 | 0.673 | 0.658 |
| 5.67° | 0.248 | 0.816 | 0.5 |  | 0.079 | 0.679 | 0.536 |

B. RT

| Flanker distance | Flanker effect | | |  | Incongruent trials | | |
| --- | --- | --- | --- | --- | --- | --- | --- |
|  | split-half (corrected) | | |  | split-half (corrected) | | |
|  | online:  test | online: retest | laboratory |  | online:  test | online: retest | laboratory |
| Mean | 0.839 | 0.837 | 0.87 |  | 0.944 | 0.938 | 0.959 |
| 0.23° | 0.691 | 0.721 | 0.855 |  | 0.869 | 0.884 | 0.931 |
| 0.91° | 0.657 | 0.768 | 0.694 |  | 0.891 | 0.893 | 0.897 |
| 1.59° | 0.518 | 0.838 | 0.75 |  | 0.832 | 0.887 | 0.93 |
| 2.95° | 0.25 | 0.406 | 0.714 |  | 0.883 | 0.881 | 0.921 |
| 4.31° | 0.396 | 0.388 | 0.619 |  | 0.895 | 0.851 | 0.909 |
| 5.67° | 0.192 | 0.392 | 0.591 |  | 0.909 | 0.839 | 0.892 |

Table S2. Split-half ICC values predicted by the Spearman-Brown prediction formula for the parameters in the linear functions.

| Parameter | Flanker effect | | |  | Incongruent trials | | |
| --- | --- | --- | --- | --- | --- | --- | --- |
|  | split-half (corrected) | | |  | split-half (corrected) | | |
|  | online:  test | online: retest | laboratory |  | online:  test | online: retest | laboratory |
| $a_{0}$ | 0.787 | 0.756 | 0.825 |  | 0.91 | 0.925 | 0.94 |
| $a_{1}$ | 0.136 | -0.428 | 0.666 |  | 0.564 | 0.209 | 0.782 |

S3. Reliability of accuracy in the SFT

Figure S1 illustrates the changes in the mean flanker effect in accuracy between sessions in the online and laboratory SFTs. The four graphs describe the data used for the calculation of the ICCs on the top row of the four left columns in Table 1A in the main text. The flanker effect is small in general, with many participants showing zero or near-zero flanker effect. Due to small variability among participants, a few participants that show relatively large flanker effect (e.g., > 0.1) can strongly bias the measures of reliability. This is most clearly shown in the online retest data, which showed the lowest reliability (ICC = 0.258).


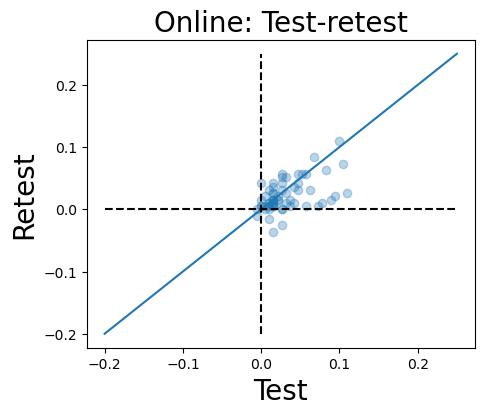

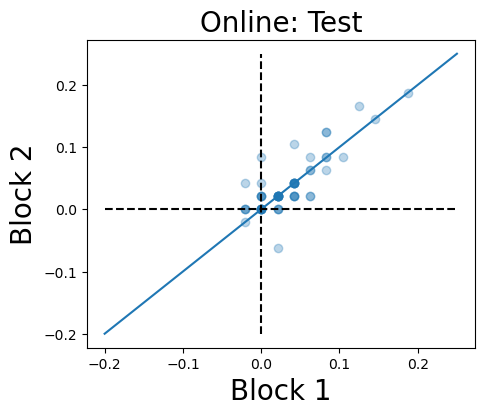

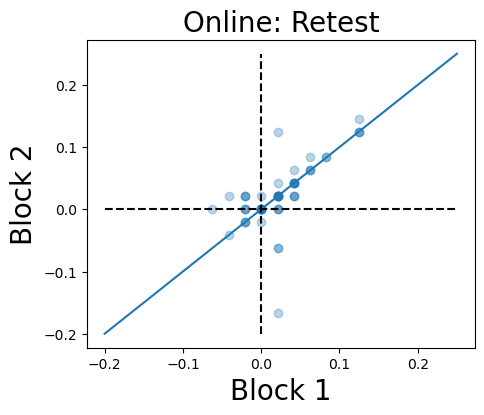

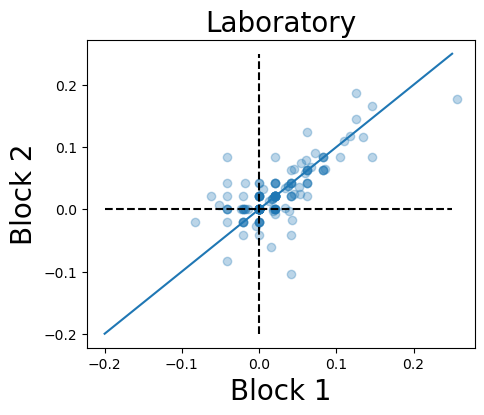


Figure S1. Scatterplots of the flanker effect in accuracy in the SFT.

S4. Additional statistics in the SFT

Table S3 below shows the standard error of the mean flanker effect and decrease in the flanker effect between sessions (i.e., practice effect) for each of the six distances in the three data sets. The standard error tended to decrease with distance in the test session. The differences among distances decreased in the retest, in part due to practice effects that reduced individual variations in the flanker effect. Statistical evidence supported practice effects at 0.23° and 2.95° in the online-only data set, but not at the other distances. The Bayes factors for practice effects were calculated by comparing the null model that assumes no practice effects with the alternative model that assumes practice effects.

Table S3. Standard errors of the mean flanker effects and practice effects in the SFT

A. Online-only data set

| Distance | Standard error of the mean: test | Standard error of the mean: retest | Practice effect |
| --- | --- | --- | --- |
| 0.23° | 5.12 ms | 4.28 ms | 25.09 ms  (BF > 100) |
| 0.91° | 4.82 ms | 3.95 ms | 2.84 ms  (BF = 0.08) |
| 1.59° | 4.52 ms | 3.67 ms | 10.82 ms  (BF = 1.64) |
| 2.95° | 3.84 ms | 3.04 ms | 13.91 ms  (BF = 4.62) |
| 4.31° | 2.74 ms | 3.48 ms | 8.02 ms  (BF = 0.38) |
| 5.67° | 3.27 ms | 3.29 ms | 3.70 ms  (BF = 0.12) |

B. Laboratory-only data set

| Distance | Standard error of the mean: test | Standard error of the mean: retest | Practice effect |
| --- | --- | --- | --- |
| 0.23° | 5.52 ms | 4.98 ms | -7.56 ms  (BF = 0.13) |
| 0.91° | 4.79 ms | 4.41 ms | -3.16 ms  (BF = 0.05) |
| 1.59° | 4.14 ms | 3.88 ms | -2.85 ms  (BF = 0.05) |
| 2.95° | 3.76 ms | 3.64 ms | 4.98 ms  (BF = 0.08) |
| 4.31° | 3.84 ms | 3.97 ms | 2.51 ms  (BF = 0.05) |
| 5.67° | 3.93 ms | 4.01 ms | 1.18 ms  (BF = 0.04) |

S5. The flanker effect in RT in blocks 1 and 2 (split-half) at each distance in the laboratory data set.


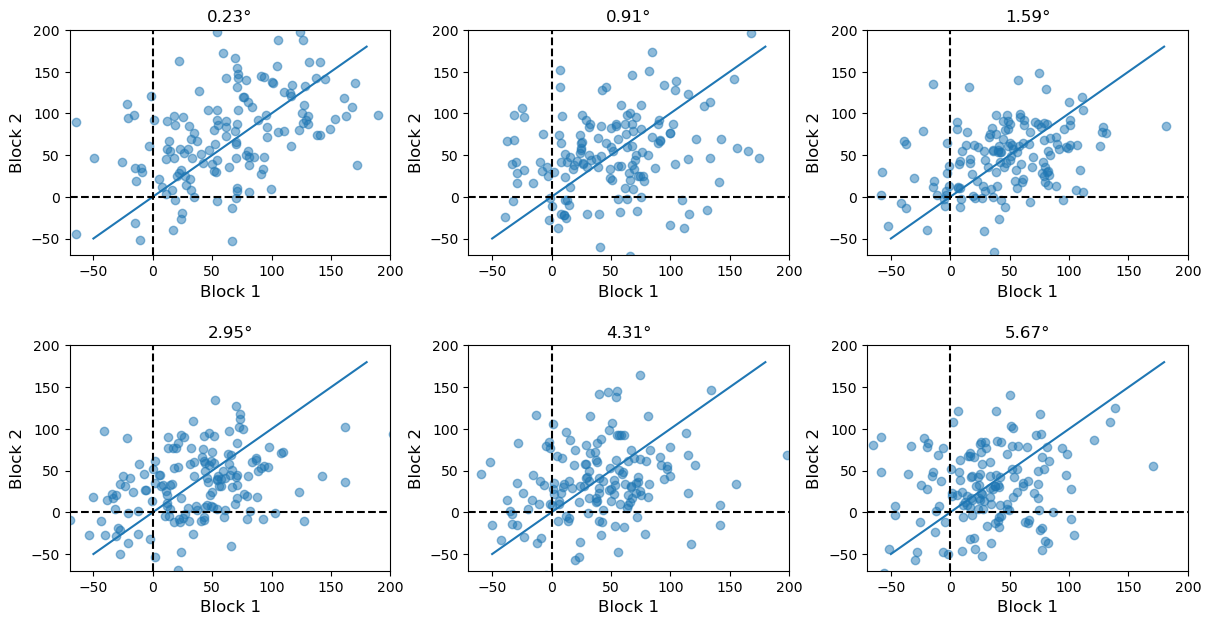


Figure S2. Scatterplots of the flanker effect (ms) in blocks 1 and 2 at each distance in the laboratory data set. Dotted lines indicate zero flanker effects. The blue lines are x=y lines.

**References**

Salvatier, J., Wiecki, T. V., & Fonnesbeck, C. (2016). Probabilistic programming in Python using PyMC3. *PeerJ Computer Science*, *2*, e55.

Wetzels, R., Grasman, R. P., & Wagenmakers, E. J. (2012). A default Bayesian hypothesis test for ANOVA designs. *The American Statistician*, *66*(2), 104-111.
